# Does the addition of vitamin E to conventional UHMWPE improve the wear performance of hip acetabular cups? Micro-Raman characterization of differently processed polyethylene acetabular cups worn on a hip joint simulator

**DOI:** 10.1590/1414-431X20209930

**Published:** 2020-08-17

**Authors:** M. Di Foggia, S. Affatato, P. Taddei

**Affiliations:** 1 Department of Biomedical and Neuromotor Sciences, University of Bologna, Bologna, Italy; 2 Medical Technology Laboratory, IRCCS - Rizzoli Orthopaedic Institute, Bologna, Italy

**Keywords:** Vitamin-E doped PE, Cross-linked PE, Standard UHMWPE, Hip simulator, Raman spectroscopy

## Abstract

In knee replacements, vitamin E-doped ultra-high molecular weight polyethylene (UHMWPE) shows a better wear behavior than standard UHMWPE. Therefore, different sets of polyethylene (PE) acetabular cups, i.e. standard UHMWPE and cross-linked polyethylene irradiated with 50 kGy and 75 kGy, were compared, at a molecular level, with vitamin E-doped UHMWPE to evaluate their wear performance after being tested on a hip joint simulator for five million cycles. Unworn control and worn acetabular cups were analyzed by micro-Raman spectroscopy to gain insight into the effects of wear on the microstructure and phase composition of PE. Macroscopic wear was evaluated through mass loss measurements. The data showed that the samples could be divided into two groups: 1) standard and vitamin E-doped cups (mass loss of about 100 mg) and 2) the cross-linked cups (mass loss of about 30-40 mg). Micro-Raman spectroscopy disclosed different wear mechanisms in the four sets of acetabular cups, which were related to surface topography data. The vitamin E-doped samples did not show a better wear behavior than the cross-linked ones in terms of either mass loss or morphology changes. However, they showed lower variation at the morphological level (lower changes in phase composition) than the UHMWPE cups, thus confirming a certain protecting role of vitamin E against microstructural changes induced by wear testing.

## Introduction

Ultra-high molecular weight polyethylene (UHMWPE) is the biomaterial used for more than twenty years as acetabular cups in the orthopedic field. Although this polymer has exceptional properties (chemical inertness, lubricity, impact and abrasion resistance), sadly oxidative degradation may decrease its mechanical properties leading to debris production and possible osteolysis and implant loosening (1,2). Prosthesis failure is mainly due to cellular reactions induced by the formation of PE debris induced by mechanical loading (3). Actually, upon wear, the surface of the cup may undergo a variation of crystallinity induced by a mechanical action: abrasion causes a decrease of the molecular weight of polymeric material and the consequent removal of the shortest macromolecular chains (4). In polymers, the crystallinity is higher if the average macromolecular chain is shorter; because of the reduced length, inter-chain interactions are lower and therefore the production of debris is higher.

The structure adopted by UHMWPE is based on long molecular chains (with an average molecular weight of 10^6^ Daltons) that fold into crystalline lamellae (10-50-nm thick and 10-50-µm long) possessing orthorhombic structure. Those domains are connected by a matrix composed of anisotropic amorphous phase and an intermediate anisotropic disordered phase (“third phase”), which is characterized by a prevailing trans-conformation and poor lateral order (5). This structural organization ensures the mechanical integrity and toughness of UHMWPE but might be undermined by the decrease in molecular weight and the increase in crystallinity, which have been reported to deteriorate UHMWPE mechanical properties (6).

Efforts have been done to solve this problem by improving the mechanical and molecular characteristics of the polymer, i.e., by radiation cross-linking. Vitamin E was introduced to solve the oxidation problem (7) and its presence should protect the radiation-cross-linked polymer from oxidation, thus avoiding re-melting (8).

The wear performance of these improved biomaterials is often evaluated using hip joint simulators. Hip wear simulation tests have been used for 40 years and they represent a powerful system to assess wear resistance before clinical use (9).

A reduction in wear rate by the addition of vitamin E to highly cross-linked PE (XLPE) compared to standard UHMWPE was observed by some preclinical *in vitro* studies (10,11). On the other hand, more contradicting results were obtained when vitamin E was added to UHMWPE. This material has attracted a certain interest thanks to its lower osteolytic and inflammatory potential compared with UHMWPE (12), its improved mechanical properties (13,14), and its higher resistance to ageing (15), delamination, and fatigue cracks that are associated with oxidation (16). Bladen et al. (12) have reported that the addition of vitamin E to non-irradiated UHMWPE did not affect the wear factor. On the contrary, Teramura et al. (17) reported that the wear volume of vitamin E-blended UHMWPE tested with a knee joint simulator was 45% lower than that of virgin UHMWPE after 5 million cycles and a similar wear rate was observed also by Schwiesau et al. (15) after ageing. Wolf et al. (18) found that vitamin E blending increased UHMWPE lifetime in oxidative conditions without leading to an enhanced wear rate or embrittlement of the material.

To gain more insight into this subject, we tested, on a hip joint wear simulator, four sets of differently processed PE acetabular cups, i.e., standard UHMWPE (hereafter STD), vitamin E-doped standard UHMWPE (hereafter VE), and highly cross-linked polyethylene irradiated with 50 kGy (hereafter XL-50) and 75 kGy (hereafter XL-75). The better wear performance reported by Teramura et al. (17) and Schwiesau et al. (15) was obtained on knee replacements, i.e., with kinematic and working loads of the implant not comparable to hip prostheses. Therefore, we asked whether the addition of vitamin E to standard UHMWPE could be a benefit also in acetabular cups and could improve wear performance compared with both UHMWPE and XLPE.

The present investigation was a continuation of a previous study (19), in which the wear behavior of the above-mentioned four sets of cups was examined together with their surface topography, cross-linking density, and oxidation state of pristine materials. Surface topography analyses revealed that worn STD and VE cups had smoother surfaces than XL-50 and XL-75 samples; VE cups showed the lowest roughness and delamination (19). In the present study, we decided to integrate the already obtained results with a molecular characterization of the materials through micro-Raman spectroscopy to link the Raman results to the surface topography data and to gain more insight into the wear mechanism. We chose Raman spectroscopy since this technique has been used by several authors (11,20,21) to evaluate the possible crystallinity changes induced by mechanical stress since it is recognized that wear involves crystallinity changes of the polymer. Moreover, Raman spectroscopy is a non-destructive technique, which allows analysis with no need for sample manipulation. This characteristic appears particularly useful because the tests can be continued under more severe conditions (i.e. after accelated ageing and upon addition of third-body wear particles). Only at the end of the following tests, will the cups be sacrificed for infrared (IR) measurements to evaluate the anti-oxidative properties of the added vitamin E.

## Material and Methods

### Biomaterials and wear simulation

Four different sets of polyethylene (PE) acetabular cups (32-mm inner × 50-mm outer diameter; six specimens for each batch) coupled with 32-mm cobalt-chromium-molybdenum (CoCrMo) femoral heads were analyzed using a hip joint simulator. Three specimens of each batch ran on the simulator following a standardized procedure (22). Another three acetabular cups for each type of material used were stored (non-loaded) in bovine calf serum to compensate for weight changes due to fluid absorption. As detailed in a previous study (19), all polyethylene cups tested in this study were machined from polymer bars of Chirulen GUR 1020 (Polymax, Adler, Italy). Cross-linked XL-50 and XL-75 acetabular cups were firstly γ-ray-irradiated with 50 and 75 kGy (±10%), respectively, and then thermally treated at 150°C (re-melted). In this way, free radicals formed during irradiation were removed. After these treatments, the cups were machined to their final shape. Similarly, VE acetabular cups were vitamin E-containing (0.1% mass) UHMWPE acetabular cups and were machined from vitamin E-blended UHMWPE bars (Polymax, Adler). The amount of vitamin E was chosen by the producer according to the results reported in the literature (23): the incorporation of 0.1% vitamin E in PE has been reported to be sufficient to stabilize PE against oxidative degradation. The STD cups were made of UHMWPE that had not received any treatment. All the cups were then subjected to ethylene oxide sterilization. All polyethylene acetabular cups were pre-soaked for four weeks in a bath of deionized water before wear tests.

The wear test was performed using a 12-station hip joint simulator (IORSynthe, Italy) for five million cycles. The test was carried out by applying the kinematic inputs and outputs simulating the complex kinematics and kinetics of the human hip in a physiological environment, running following ISO 14242-3 axial load control (24). The lubricant used was 25% (vol) newborn calf serum balanced with distilled water, with 0.2% (mass) sodium azide to retard bacterial growth and 20 mM EDTA to minimize precipitation of calcium phosphate. The mass loss of the cups was measured at the end of the test using a semi-micro balance (Sartorius Cubis Mse 225 S-000-DU, Germany, resolution 0.01 mg), and was corrected by acetabular soak control.

### Micro-Raman spectroscopy

The central area of the articulating surface of the STD, XL-50, XL-75, and VE acetabular cups was analyzed by micro-Raman spectroscopy after wear testing and compared with unworn non-loaded components as control samples. The central area proved to be the most worn based on previous studies (25); in the area where spectra were taken, topographical features analogous to those reported in a previous study (19) were observed. Raman spectra were acquired in the areas that showed the most significant surface topography change.

Micro-Raman spectra were obtained using an NRS-2000C (Jasco International Co. Ltd., Japan) instrument with a microscope of 50× magnification. All the spectra were recorded in back-scattering conditions with 5 cm^-1^ spectral resolution using the 532 nm green diode-pumped solid-state laser driver (RgBLase LLC, USA) with a power of about 20 mW. A 160 K cooled digital charge-coupled device (Spec-10: 100B, Roper Scientific Inc., USA) was used as a detector.

A confocal pinhole with an aperture diameter of 200 μm was placed in the optical circuit to obtain signals from a limited in-depth region. Micro-Raman spectra were recorded on twelve different points on each cup; each spectrum is the average of four accumulations.

The Raman spectrum of PE is well characterized (Supplementary Figure S1) and various studies have aimed at identifying marker bands of PE morphology and correlating their intensity to the polymer crystallinity (5,26,27).

The fractions of orthorhombic (α_o_), amorphous (α_a_), and intermediate anisotropic disordered (i.e., a “third phase” with a prevailing trans-conformation of the chains, which have lost their lateral order) (α_b_) phases were calculated from the areas of selected Raman bands, according to the equations proposed by Strobl and Hagedorn (5) and also used by other authors (26,27):

$$\alpha_{o}=\frac{A_{1416}}{0.46\times A_{1295+1305}}$$ (1)

$$\alpha_{a}=\frac{A_{1085}}{0.79\times A_{1295+1305}}$$ (2)

$$\alpha_{b}=1–(\alpha_{o}+\alpha_{a})$$ (3)

where A_1416_ and A_1085_ are the areas of the Raman bands at about 1416 and 1085 cm^-1^ respectively, and A_1295+1305_ is the area of the internal conventional (i.e., independent of chain conformation) band group. The A_1085_ band area was determined after a curve-fitting analysis of the 1040-1110 cm^-1^ range using a fitting software (Fityk 1.3.1, GNU GPL License by M. Wojdyr). Curve fitting was performed on the original spectra after baseline correction, using the Levenberg-Marquardt algorithm. The Raman components are described as linear combinations of Gaussian and Lorentzian functions.

The above-reported equations come from a comparison between the Raman spectra of PE samples with a different crystallinity degree with their wide and small angle x-rays diagrams. The mass fractions involved in the three phases can be derived directly from the integral intensities of characteristic bands without an additional calibration procedure (5). The calculated results show a good linear correlation with the crystallinities derived from other methods, i.e., differential scanning calorimetry (DSC) and X-ray diffraction (XRD) measurements (28).

Concerning the calculation of the α_a_ amorphous content, an earlier study (29) has reported the band at 1305 cm^-1^ as more reliable for this purpose. However, as previously observed (11), the amorphous content calculated through the 1305 cm^-1^ band is higher than that calculated through the 1085 cm^-1^ band. In this study, we have preferred to use the latter band since using the former, negative intermediate phase contents were obtained.

$$\mathrm{all–trans}=\frac{A_{1130}}{0.80\times A_{1295+1305}}$$ (4)

$$\mathrm{ortho–trans}=\frac{1.78\times A_{1416}}{A_{1130}}$$ (5)

The fractions of all-trans sequences and all-trans sequences in an orthorhombic environment (ortho-trans) were evaluated through the equations developed by Lagaron et al. (28) and Naylor et al. (27):

where A_1130_ is the area of the Raman band at about 1130 cm^-1^, which has been associated with all-trans C-C bonds located both in the crystalline and amorphous phases. Actually, in the crystalline phase, the carbon skeleton has a planar zigzag structure, with the C-C bonds in the all-trans conformation; on the contrary, the amorphous phase is composed of different sequences of trans and gauche bonds. The area of the bands mentioned in the preceding text was determined using commercial software (Spectra Analysis, Jasco Corporation, Japan).

At this moment, it must be stressed that the band at 1130 cm^-1^ is affected by molecular chain orientation, which can bias the results of all-trans and ortho-trans fractions. Lagaron et al. (28) have shown that molecular orientation can also affect the Strobl and Hagedorn formalism. To verify that the polarized incident laser did not affect the Raman intensity detected from the samples, the A_1130_/A_1065_ band area ratio was determined to evaluate the occurrence of orientation upon wear testing, according to previous studies (28,30). The bands at 1130 and 1065 cm^-1^ have different vibrational symmetries (30) and, if the molecules are oriented in a preferred direction, the 1130 cm^-1^ band has been reported to become stronger than the 1065 cm^-1^ band. Additionally, some selected worn acetabular cups (i.e., those that underwent the highest strengthening in the 1130 cm^-1^ band upon wear) were analyzed at different laser polarization; the rotation of the polarization plane of the incident radiation was obtained with a half-wave plate. According to Porto's nomenclature, the scattering geometry can be described by a four-letter notation: A(BC)D, where A, B, C, and D stand for one of the axes of the external reference frame, X, Y, or Z. A is the direction of the laser beam, B the polarization of the laser, C the direction of the polarization analyzer, and D the direction of view (the direction for the collection of the scattered intensities). Spectra were recorded in X(YY)X and X(ZY)X optical configurations.

Statistical analysis on Raman data was performed with R statistical software (version 3.5.3; GNU GPL license). The sample size considered for statistical analysis was n=3 (means of 3 samples for each batch). The data had a non-Gaussian distribution, so a non-parametric Kruskal-Wallis test was used to determine the statistical significance (set at P<0.05), and a Dunn-Bonferroni post-hoc analysis was performed for any dependent variable for which the Kruskal-Wallis test was significant. The Kruskal-Wallis test does not compare means but is based on ranks and was used to verify if the rank means were different. Nevertheless, we report the data as average values with their associated standard deviation (SD), for better readability.

## Results

### Mass loss

**Figure 1:**
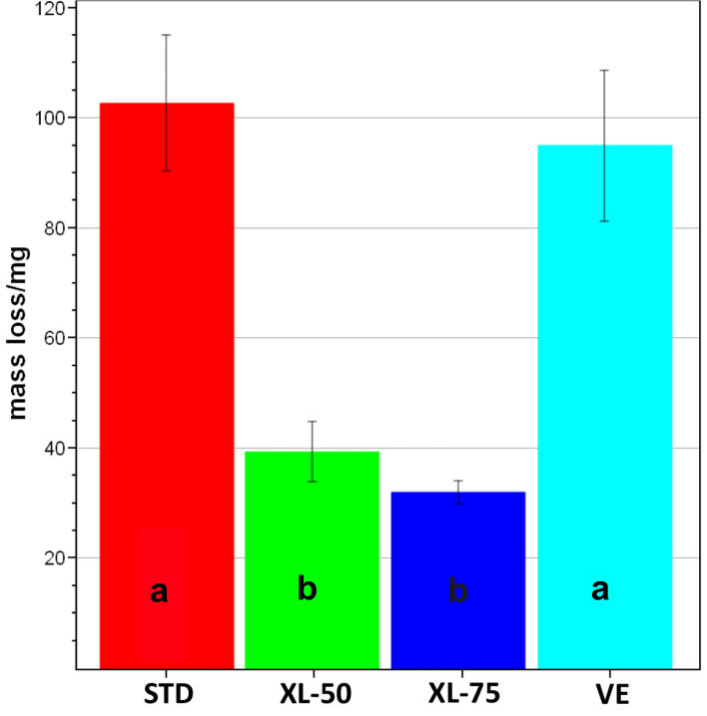
Average (±SD) mass loss of the four sets of acetabular cups at the end of the wear test. Different letters indicate a significant difference among treatments in a *post hoc* Dunn-Bonferroni test (P<0.05). STD: standard polyethylene; XL-50: cross-linked polyethylene irradiated with 50 kGy; XL-75: cross-linked polyethylene irradiated with 75 kGy; VE: vitamin E-doped standard polyethylene.

Figure 1 shows the average mass loss after the wear test for the four sets of acetabular cups. As can be easily seen, cups can be divided into two homogenous groups, statistically different between each other: the first characterized by a higher mass loss, about 100 mg (STD and VE), and the second by a significantly lower mass loss, comprised between 30 and 40 mg (XL-50 and XL-75).

### Micro-Raman spectroscopy: unworn samples

**Figure 2:**
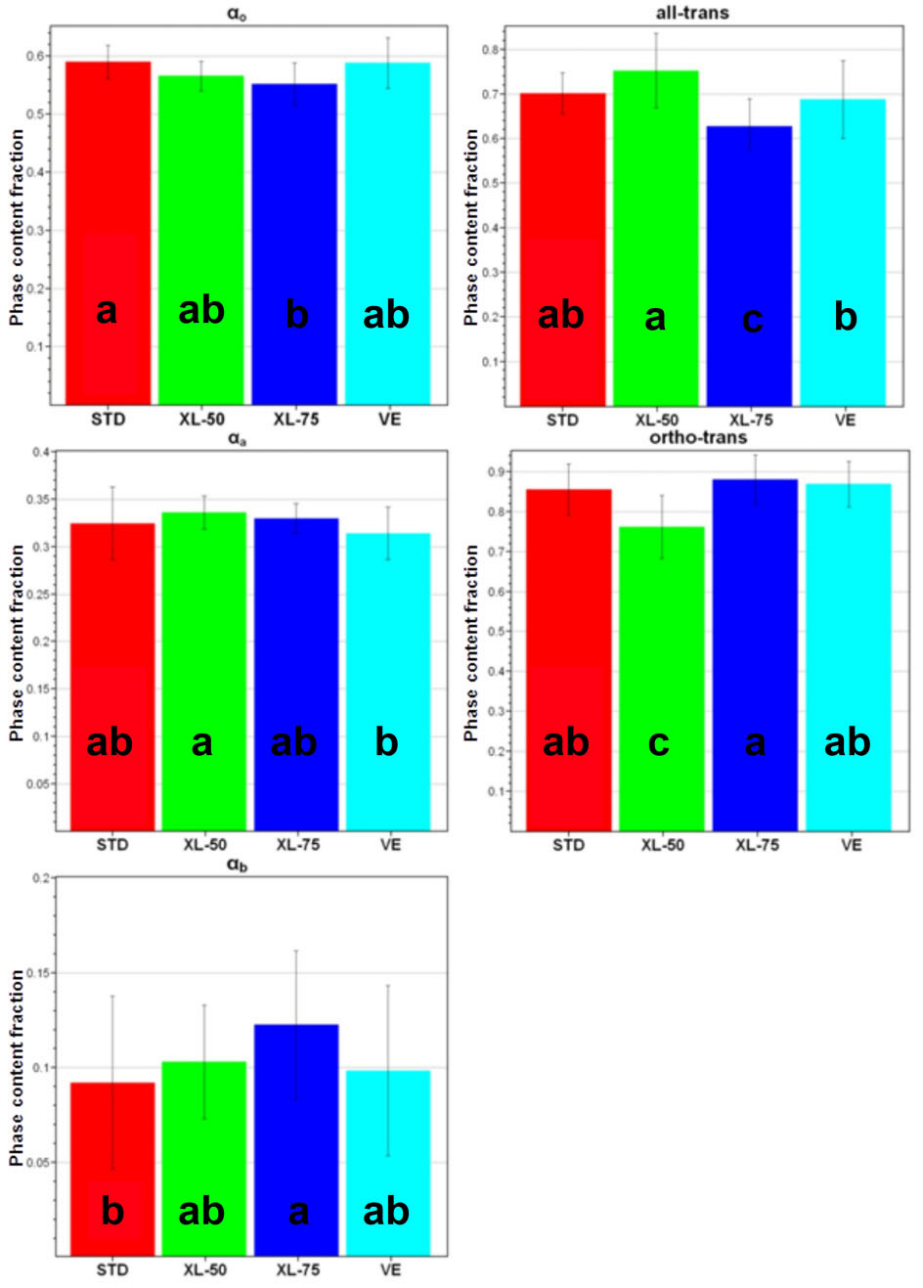
Values (average±SD) of α_o_, α_a_, α_b_, all-trans, and ortho-trans contents, as obtained from the spectra recorded on the unworn STD, XL-50, XL-75, and VE acetabular cups. Different letters indicate a significant difference among treatments in a *post hoc* Dunn-Bonferroni test (P<0.05). STD: standard polyethylene; XL-50: cross-linked polyethylene irradiated with 50 kGy; XL-75: cross-linked polyethylene irradiated with 75 kGy; VE: vitamin E-doped standard polyethylene.

**Figure 3:**
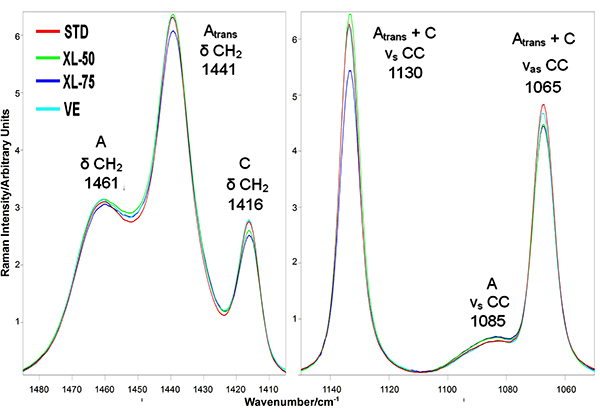
Average micro-Raman spectra of STD, XL-50, XL-75, and VE unworn acetabular cups in the most representative spectral ranges. The spectra are normalized to the A_1295+1305_ area. STD: standard polyethylene; XL-50: cross-linked polyethylene irradiated with 50 kGy; XL-75: cross-linked polyethylene irradiated with 75 kGy; VE: vitamin E-doped standard polyethylene.

The data reported in Figure 2 allowed to examine quantitatively the differences among the four sets of unworn acetabular cups, whose average micro-Raman spectra are reported in Figure 3 in the most representative spectral ranges.

The orthorhombic α_o_ content of STD was significantly higher than that of XL-75 (as qualitatively observable from the relative intensity of the main marker band of this phase at 1416 cm^-1^ (5), Figure 3); on the other hand, the intermediate phase α_b_ content was significantly lower in STD than in XL-75. XL-50 showed the highest amorphous α_a_ content (i.e. the highest relative area of the band at 1085 cm^-1^ (5)), which was statistically different from VE cups. The all-trans content had a more complex behavior: this parameter, which reflects the relative intensity of the 1130 cm^-1^ band (Figure 3), was significantly higher in XL-50 compared to all the other unworn cups, while the other cross-linked set (i.e., XL-75) showed the lowest content, which was found to be statistically different from that of STD. VE cups had an intermediate all-trans content that was statistically different from XL-50 and XL-75 cups, but not from STD. The ortho-trans content showed an opposite trend: XL-50 showed the lowest content, while XL-75 the highest. STD and VE cups proved to be both statistically different from XL-50 cups. These findings reflect the trend of the micro-Raman spectra of the unworn cups (Figure 3): XL-50 and XL-75 had respectively the highest and the lowest intensity of the band at 1130 cm^-1^ that, as can be easily seen from equations (4) and (5), is directly proportional to the all-trans content and inversely proportional to the ortho-trans content.

### Micro-Raman spectroscopy: worn samples

**Figure 4:**
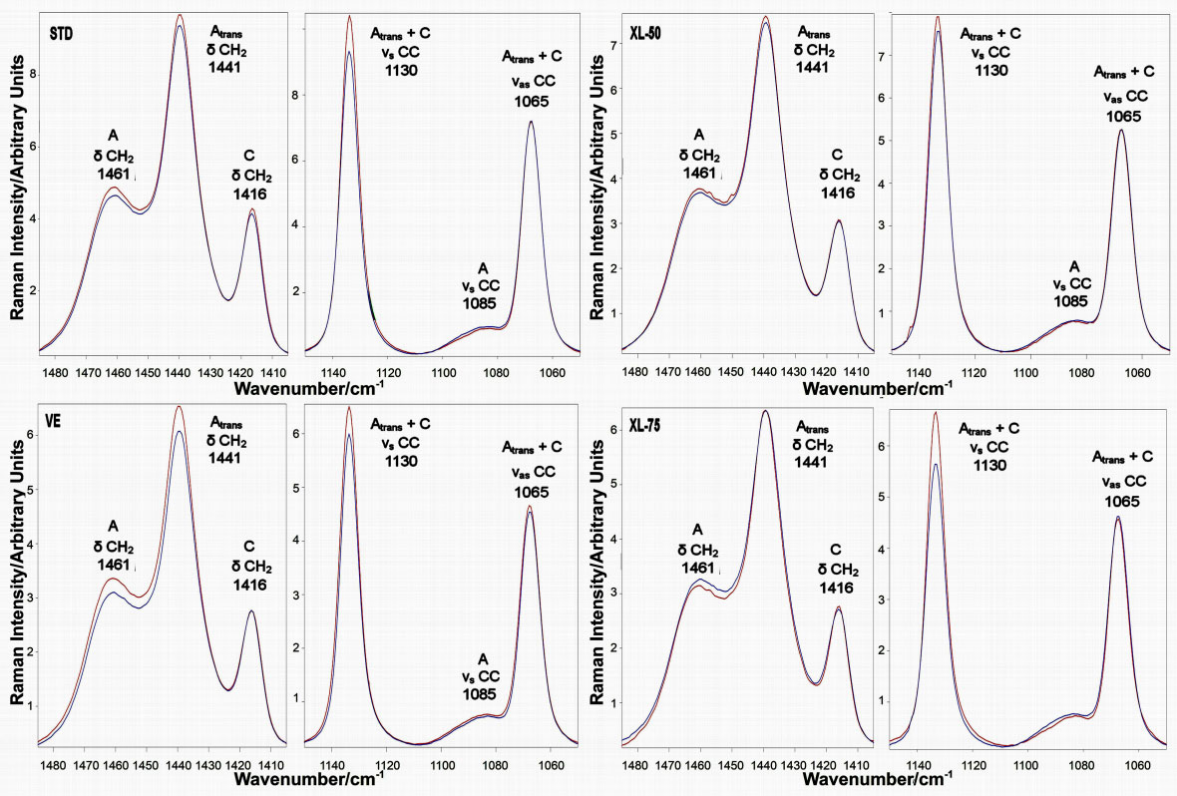
Average micro-Raman spectra of STD, VE, XL-50, and XL-75 unworn acetabular cups (blue) and after the wear test (red). The spectra are normalized to the A_1295+1305_ area. STD: standard polyethylene; XL-50: cross-linked polyethylene irradiated with 50 kGy; XL-75: cross-linked polyethylene irradiated with 75 kGy; VE: vitamin E-doped standard polyethylene.

The average micro-Raman spectra recorded on the four sets of cups at the end of the wear test are shown in Figure 4, together with the spectra of the unworn samples. In general, the average micro-Raman spectra of the cross-linked acetabular cups (XL-50, in particular) showed less significant variations upon wear than the non-cross-linked ones (i.e., STD and VE). Actually, in the STD set a detectable change in intensity of both the bands at 1416 cm^-1^ (assigned to orthorhombic PE) and 1085 cm^-1^ (assigned to amorphous PE) was observed; the former strengthened, while the latter weakened. The other sets of acetabular cups underwent less significant variations in these spectral ranges. The most significant changes were observed in the relative intensity of the band at 1130 cm^-1^ (C-C stretching region), which showed a general increase in all worn cups. As stressed in the experimental section, the latter spectral feature is sensitive to molecular chain orientation, which can also affect the results of all-trans and ortho-trans fractions, as well as the Strobl and Hagedorn formalism (28). To verify that the polarized incident laser did not affect the Raman intensity detected from the samples, the A_1130_/A_1065_ band area ratio was determined to evaluate the occurrence of orientation upon wear testing, according to previous studies (28,30). The corresponding data are reported in Supplementary Figure S2; as can be easily seen, no statistically significant change was observed in the A_1130_/A_1065_ ratio, suggesting that, upon wear testing, the acetabular cups remained isotropic and molecular orientation did not occur. Additionally, some selected worn acetabular cups (i.e., those that underwent the highest strengthening in the 1130 cm^-1^ band upon wear) were analyzed at different laser polarizations. The spectra recorded in X(YY)X and X(ZY)X optical configurations on STD and VE cups are reported in Supplementary Figures S3 and S4. As can be seen, the spectra recorded under different laser polarizations were practically coincident, confirming that testing under the multi-directional wear conditions typical of the hip joint simulator did not induce any molecular chain orientation.

**Figure 5:**
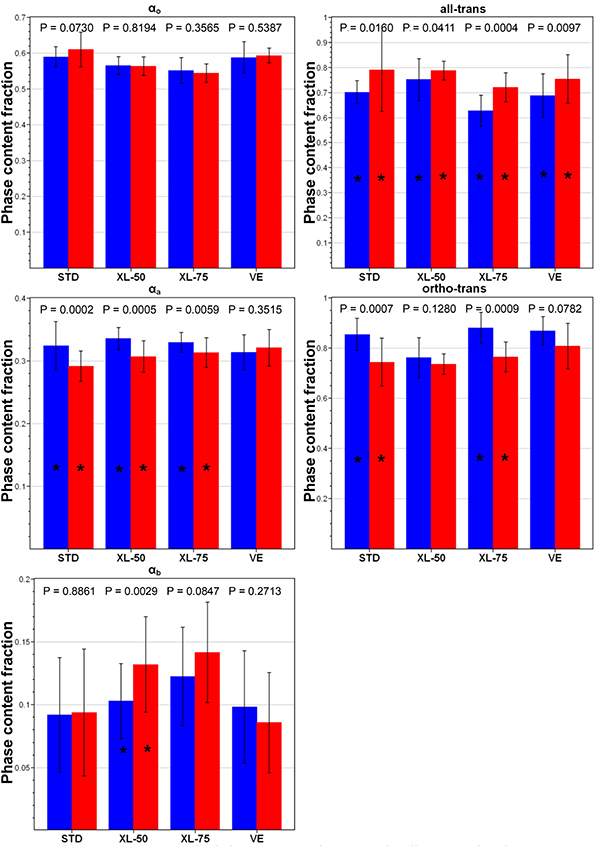
Values (average±SD) of α_o_, α_a_, α_b_, all-trans, and ortho-trans contents, as obtained from the spectra recorded on the unworn STD, XL-50, XL-75, and VE acetabular cups (blue bars) and after the wear tests (red bars). The asterisk (*) indicates a significant difference of the values before and after wear in a post hoc Dunn-Bonferroni test (P<0.05). STD: standard polyethylene; XL-50: cross-linked polyethylene irradiated with 50 kGy; XL-75: cross-linked polyethylene irradiated with 75 kGy; VE: vitamin E-doped standard polyethylene.

The quantitative data reported in Figure 5 allowed more insight into the qualitative trends observed from the spectra. The α_o_ orthorhombic content was shown to be not significantly affected by wear (only STD acetabular cups were quite close to significance). On the contrary, a significant reduction of α_a_ amorphous phase was observed in all cups after wear (except for VE cups), together with a general increase of the intermediate (α_b_) phase (significant in XL-50 cups and again VE cups showing the opposite trend).

The all-trans content increased in all cups upon wear: this increase was found to be significant in all sets. The opposite trend was observed in the ortho-trans content: this parameter significantly decreased upon wear in STD and XL-75 cups.

**Figure 6:**
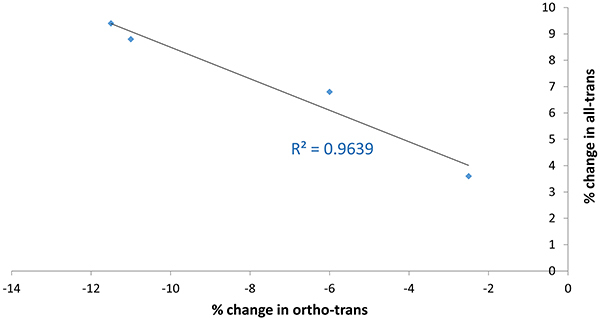
Trend of the percent change observed upon wear test in the all-trans content of the analyzed acetabular cups as a function of the percent change in the ortho-trans content. Average data for each set of acetabular cups are reported.

The latter results were confirmed by the trend of the graphs reported in Figure 6 and Supplementary Figures S5-S9: the percent change in the all-trans content was linearly related to that in the ortho-trans content, by considering either average data corresponding to the four sets of acetabular cups (Figure 6) or data on the single analyzed cups considered as a whole (Supplementary Figure S5) or divided for material type (Supplementary Figures S6-S9).

**Figure 7:**
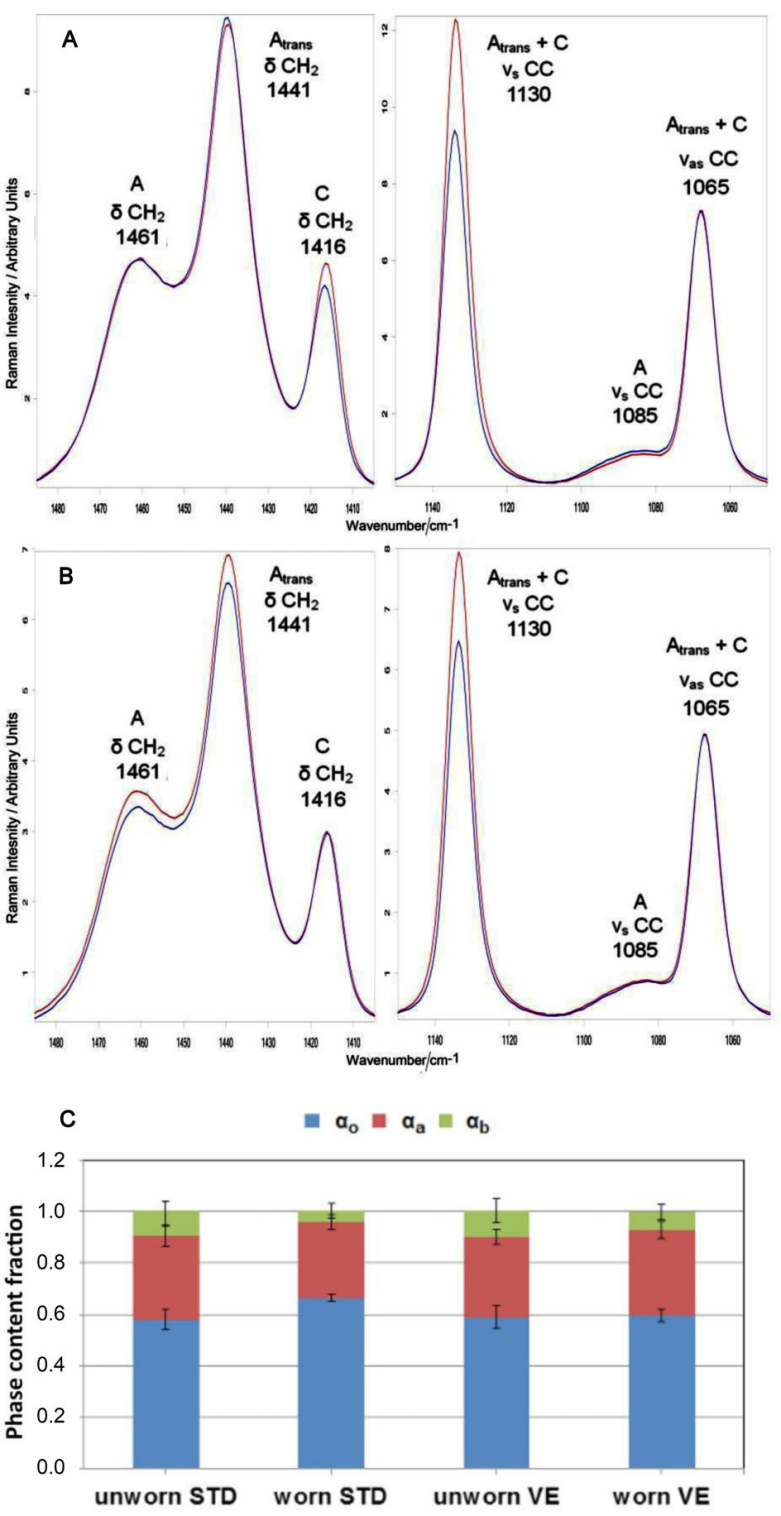
Average micro-Raman spectra of single selected STD (A) and VE (B) acetabular cups after (red) wear testing. The spectra of unworn cups are reported for comparison (blue). The chosen samples underwent a similar mass loss (about 110*-*120 mg) upon wear test. The spectra are normalized to the A_1295+1305_ area. C, Values (average±SD) of α_o_, α_a_, and α_b_ contents, as obtained from the spectra recorded on the same acetabular cups. STD: standard polyethylene; VE: vitamin E-doped standard polyethylene.

To gain more insight into possible differences in wear mechanism between STD and VE acetabular cups, the average micro-Raman spectra of two selected samples belonging to the two sets and characterized by a similar mass loss upon simulator testing (about 110-120 mg, i.e., the highest in their respective sets) were compared and are reported in Figure 7.

As can be easily seen, the two chosen samples underwent different changes upon wear testing. The STD cup underwent an almost significant (P=0.0730, reported in Figure 5) increase in the α_o_ content, from 0.59 (SD: 0.03) to 0.62 (SD: 0.05), Figure 7C), as also revealed by the strengthening of the 1416 cm^-1^ band (Figure 7A). The micro-Raman data showed that the increase in crystallinity occurred at the expense of the amorphous phase and interphase, which both decreased their content upon wear (the former to a lower extent than the latter, Figure 7C). Such behavior was not observed for the selected VE cup; upon wear testing, it did not undergo any significant increase in crystallinity (Figure 7B and C). At the same time, it underwent a slight increase in the amorphous content (Figure 7C), as also revealed by the strengthening of the bands at 1441 and 1461 cm^-1^ (Figure 7B), at the expenses of the interphase (Figure 7C). The all-trans and ortho-trans contents changed to the same extent in the two selected samples: the former increased (as also shown by the strengthening of the 1130 cm^-1^ band, Figure 7A and B) and the latter decreased.

## Discussion

The main purpose of this work was to characterize the *in vitro* wear performance, on a molecular scale, of four sets of differently processed PE acetabular cups coupled with CoCrMo femoral heads. Specifically, we investigated whether the sole addition of vitamin E to conventional UHMWPE could improve its wear performance also in comparison with highly cross-linked PE. In this comparative study, we chose to test acetabular cups rather than knee prostheses, since previous studies (15,17) have already reported a better wear behavior of VE-added UHMWPE in knee replacements, while no analogous study, to the best of our knowledge, has been performed on hip prostheses. Moreover, it must be observed than in our investigation we have also included cross-linked polyethylene, whose use in knee arthroplasty is much more limited due to recurrent concerns about its suitability.

As reported above, the present paper should be considered the continuation of a previous study by Affatato et al. (19) that described the initial characterization of the same sets of acetabular cups, focusing on surface topography and roughness measurements. Moreover, oxidation degree (by IR spectroscopy), cross-linking density, and crystallinity were measured only on the pristine samples: cross-linking density was found to correlate with the reduced mass loss of cross-linked samples, while IR spectra did not show any difference in the oxidation state of the cups. The cross-linked worn cups showed an increased roughness, which could be an index of fatigue wear. Since the study left some open questions, we asked whether Raman spectroscopy could give a molecular insight into the differences observed through the previous techniques: the appeal of this technique also relies on its non-invasiveness, a very useful property because of the further continuation of the wear study on the same cups.

Unworn control and worn acetabular cups were analyzed by micro-Raman spectroscopy to gain insight into the effects of wear on the microstructure and morphology of PE; at the same time, macroscopic wear was evaluated through mass loss measurements. Three samples for each set were tested and twelve Raman spectra were recorded in the center of each cup, as detailed in the experimental section. This number of samples proved suitable since no significant differences (P>0.05) were observed between the specimens belonging to the same set in each of the spectroscopic parameters (Supplementary Table S1).

The micro-Raman characterization of unworn cups showed that the samples may be divided into two groups: the first one comprised STD and VE, the other XL-50 and XL-75. Actually, the average spectra of unworn STD and VE were practically coincident and no significant differences in the spectroscopic markers were observed between these sets of samples. This result showed that the addition of vitamin E did not alter the chemical characteristics of STD. Different results were observed upon the incorporation of vitamin E (31) and vitamin C (32) into standard UHMWPE. The main differences were observed between unworn non-cross-linked and cross-linked acetabular cups. Specifically, in agreement with previous studies (22), the XL-75 unworn samples showed a significantly lower α_o_ orthorhombic content. This behavior may be explained by considering that constraints imposed on the chains due to the formation of a cross-linked network structure reduce the degree of order and thus of crystallinity.

Mass loss measurements reflected these trends; actually, the acetabular cups can be divided into the same two groups mentioned above. The highest wear rate was observed for the STD cups, followed closely by the VE ones, whereas the wear rates of XL-50 and XL-75 were significantly lower but close to each other.

By the late 1990s, substantial improvement in the wear resistance of UHMWPE was obtained by introducing the cross-linking process. McKellop et al. (1) found that the process of cross-linking highly improved wear resistance, and the wear rate decreased markedly with increasing radiation dose, reaching a reduction of 87% for the cup irradiated at 95 kGy compared to the one irradiated at 33 kGy. In a previous study (22), we found that the wear of conventional UHMWPE was forty times higher than for cross-linked polyethylene (XLPE), testing these materials against deliberately scratched CoCrMo femoral heads.

Since cross-linking is known to increase the abrasion resistance, this explains the reduced wear observed also in the present wear test for cross-linked samples compared to the STD ones. On the contrary, the addition of vitamin E, which is intended as a stabilizer against oxidation, did not induce any significant modification in wear properties compared to STD, from a macroscopic point of view (i.e., mass loss measurements, Figure 1). However, from a morphological point of view, this is only partially true. The average spectroscopic markers showed more significant changes in the STD set than in the VE set (Figure 5): in STD cups, the contents of three phases (i.e., α_a_, all-trans, and ortho-trans) were significantly affected by wear, while VE cups showed a significant variation only in the all-trans content. This trend is even more evident by comparing two selected acetabular cups, which showed a similar mass loss that was the highest in their respective groups (Figure 7); as can be easily seen, the STD worn cup underwent an increase in the α_o_ orthorhombic content, which was not observed in the VE cup. Such an increase was not observed in either XL-50 or XL-75 upon wear, confirming the results previously reported (22). The increase in the orthorhombic content (α_o_) has already been observed in several PE articular prostheses upon either *in vitro* testing or *in vivo* service (23,33,34) and might be explained according to previous studies (34).

The cups that lost the highest mass (i.e., STD) underwent the highest increase in the orthorhombic content and the lowest change in the third phase content. This behavior suggested that in STD the orthorhombic phase increased at the expense of the amorphous phase, which was found to significantly decrease upon wear. On the contrary, in the cross-linked cups, no significant changes in the α_o_ orthorhombic content were observed and a different wear mechanism was found to operate: the third phase content increased at the expense of the amorphous phase content (Figure 5). Despite these differences in microscopic wear mechanisms, a common behavior for all sets of cups was observed: as observable in Figures 4, 5, and 6, all the samples underwent an increase in the all-trans content upon wear, i.e., strengthening of the 1130 cm^-1^ band. All of them underwent a rearrangement of the molecular chains. Actually, it has been shown computationally that as few as 10 consecutive C−C all-trans conformations will contribute intensity to the 1130 cm^-1^ band (35). Recently, Migler et al. reported that Raman spectroscopy can detect non-crystalline consecutive all-trans chain segments in a pre-crystalline stage before forming the orthorhombic state (36). They have observed that the 1130 cm^-1^ band is sensitive to non-crystalline all-trans sequences, which might develop in the amorphous phase during cold drawing due to tie chains, for instance. It must be stressed that the increase in the all-trans content upon hip simulator testing was observed independently of the change in the α_o_ orthorhombic content (Figure 5). This result is in agreement with a previous study (37), which reported a similar trend upon plastic deformation of cold-drawn polyethylene, causing chain stretching.

Figure 6 shows that the samples that underwent the highest increases in all-trans content were characterized by the highest decreases in ortho-trans content. This trend was obtained also by graphing the data obtained on single acetabular cups as a whole or divided for the type of PE material. Interestingly, good linear trends with comparable slope values were obtained in all sets of acetabular cups (Supplementary Figures S5-S9), although we are aware that by graphing the data corresponding to each set of the acetabular cup, few points are present in each graph.

The opposite trend of the all-trans and ortho-trans content upon wear may be explained with the mathematical form of equations (4) and (5), which both contained the area of the band at 1130 cm^-1^, A_1130_, i.e., the spectroscopic parameter that showed the most significant increase upon wear (Figure 4): in the former equation, A_1130_ is at the numerator, in the latter at the denominator. Moreover, with regards to equation (5), the numerator (i.e. the area of the band at 1416 cm^-1^) remained nearly constant upon wear, thus it is not surprising that the ortho-trans content decreased upon wear, as an effect of the increased A_1130_. On the other hand, as recalled above, the area of the band at 1130 cm^-1^ has a contribution from the all-trans sequences of all PE phases; therefore, its increase (Figure 6) indicated that in the sets of cups analyzed, the chain conformation was deeply affected by wear mostly in the amorphous phase.

To sum up the previous findings, it is possible to affirm that the average spectroscopic markers showed more significant changes in the STD set than in the VE set (Figure 5): in STD cups, the contents of three phases (i.e., α_a_, all-trans, and ortho-trans) were significantly affected by wear, while VE cups showed a significant variation only in the all-trans content. Specifically, the STD worn cup underwent an increase in the α_o_ orthorhombic content, which was not observed either in VE or XL-50 and XL-75.

These differences reflected the trend of the surface morphological data reported by Affatato et al. (19) on the same sets of acetabular cups; to make clearer this correlation, Supplementary Table S2 shows the statistical analysis of the parameters measured through Raman spectroscopy on the worn cups. The authors observed an increased rough surface with signs of delamination and incipient cracks in cross-linked PE cups compared to STD and VE samples. These markers of increased fatigue wear can be linked to the lower crystallinity (α_o_) measured for both XL-50 and XL-75 samples. A decrease in crystallinity has been often associated with a decrease in fatigue resistance (38,39). On the other hand, the smoother surface displayed by the STD and VE cups suggested an abrasive wear occurrence that led to a setting-in phase of the non-cross-linked polyethylene surfaces to the CoCrMo counter body. The contour images of VE cups presented an almost smooth surface with no signs of depth wear or delamination (19); on the contrary, STD cups were characterized by more frequent and larger grooves than VE, with signs of delamination along the border of the scratches. The lowest surface roughness and delamination observed by Affatato et al. (19) for VE cups may be related to its highest ortho-trans content, together with low all-trans content.

Because of the obtained results, it may be affirmed that the addition of vitamin E to STD acetabular cups played a certain protecting role against morphology changes induced by wear testing, although no significant influence of this antioxidant on the wear behavior has been revealed, differently from knee prostheses. This is not surprising because according to Wang et al. (40), the loading conditions for the knee and the hip are substantially different (uniaxial and with lack of conformation resulting in high sub-surface shear stresses in the knee *versus* multiaxial and with high conformation resulting in plastic deformation at individual contact points in the hip).

Further studies in more severe conditions, according to previous investigations (11), are planned to gain more insight into this subject and assess possible differences between the sets of cups. Under more critical conditions, we expect to get an even clearer understanding of the effect of vitamin E. For this purpose, it is interesting to recall that Bladen et al. (12) found no significant differences between the wear rates of conventional and VE-added UHMWPE when tested using a pin-on-plate wear simulator against a smooth counterface; however, they reported a better wear behavior for the VE-doped samples when tested against rough surfaces. The study of oxidative stress on worn cups through IR spectroscopy, of paramount importance to investigate the incidence of fatigue crack, was planned in the continuation of the wear testing, due to the destructive nature of IR measurements.

## Supplementary Material

Click here to view [pdf].
